# Amitriptyline for the prevention of post herpetic neuralgia: study protocol for the ATHENA study

**DOI:** 10.1093/skinhd/vzaf002

**Published:** 2025-02-14

**Authors:** Sian Wells, Stephanie J MacNeill, Yumeng Liu, Anna Gilbertson, Hazel Everitt, Oliver van Hecke, Jonathan Banks, Sophie Rees, Rebecca Kandiyali, Kirsty Garfield, Lorelei Hunt, Ioana Fodor, Vikki Wylde, Robert Johnson, Alastair D Hay, Anthony E Pickering, Matthew J Ridd

**Affiliations:** Bristol Trials Centre, University of Bristol, Bristol, UK; Bristol Trials Centre, University of Bristol, Bristol, UK; Bristol Trials Centre, University of Bristol, Bristol, UK; Centre for Applied Excellence in Skin & Allergy Research, University of Bristol, Bristol, UK; Primary Care Research Centre, University of Southampton, Southampton, UK; Department of Public Health and Primary Care, Ghent University, Ghent, Belgium; NIHR Applied Research Collaborative West (NIHR ARC West), University Hospitals Bristol NHS Foundation Trust, Bristol, UK; Bristol Trials Centre, University of Bristol, Bristol, UK; Centre for Health Economics Warwick (CHEW), Warwick Medical School, University of Warwick, Warwick, UK; Bristol Trials Centre, University of Bristol, Bristol, UK; Centre for Applied Excellence in Skin & Allergy Research, University of Bristol, Bristol, UK; Centre for Applied Excellence in Skin & Allergy Research, University of Bristol, Bristol, UK; Musculoskeletal Research Unit, University of Bristol, Bristol, UK; School of Physiology, Pharmacology & Neuroscience, University of Bristol, Bristol, UK; Centre for Academic Primary Care, University of Bristol, Bristol, UK; School of Physiology, Pharmacology & Neuroscience, University of Bristol, Bristol, UK; Centre for Applied Excellence in Skin & Allergy Research, University of Bristol, Bristol, UK

## Abstract

**Background:**

Post herpetic neuralgia (PHN) is the most common complication of herpes zoster, also known as shingles. Amitriptyline has been postulated to prevent PHN. The objective is to determine whether prophylactic low-dose amitriptyline prevents PHN in patients newly diagnosed with shingles.

**Methods:**

This is a multicentre, individually randomized, pragmatic, placebo-controlled superiority trial with health economic analysis and nested qualitative study. Patients with new-onset shingles are screened by treating clinicians in participating general practitioner surgeries. Key eligibility criteria are age ≥50 years, ≤6 days since rash onset and not already taking (and no contraindication to) amitriptyline. Participants are randomized 1:1 to amitriptyline 10 mg or matched placebo tablets (dose escalated as tolerated to a maximum of three tablets daily for 70 days). Resource-use data (including health, social and informal care, personal expenses and usual activities) are collected from electronic medical records and participant questionnaires. A sample of recruitment conversations are audio-recorded and interviews conducted with recruiters and patients, including those who decline to participate or who withdraw from the trial.

**Discussion:**

The primary outcome is the presence of PHN (≥3/10 worst pain on Zoster Brief Pain Inventory) at 90 days after rash onset. Primary health economic analyses will present cost per case of PHN prevented and cost per quality-adjusted life year. Qualitative data will be analysed to optimize trial delivery and to aid interpretation and implementation of the trial findings. This is the largest trial to date to evaluate the clinical/cost-effectiveness and acceptability of prophylactic low-dose amitriptyline for the prevention of PHN.

**Protocol registration:**

EudraCT 2021-001101-78 and ISRCTN14490832.

## Teaser

The ATHENA study is the largest study in the world to investigate whether prophylactic low-dose amitriptyline prevents post herpetic neuralgia, the most common complication of herpes zoster (shingles). This protocol paper describes the design and conduct of this multi-centre, individually randomized, pragmatic, placebo-controlled superiority trial in primary care with health economic analysis and nested qualitative study.

**What is already known about this topic?**

- Post herpetic neuralgia (PHN), the most common complication of herpes zoster (shingles), is difficult to treat.
- While vaccination reduces the incidence of shingles and, consequently, PHN, there are no known interventions that reduce the risk of the development of PHN once shingles has developed.
- One small trial suggested that low-dose amitriptyline might prevent the progression from shingles to PHN, but better evidence is needed.

**What does this study add?**

- This is the largest randomized placebo-controlled trial designed to answer the research question of whether prophylactic low-dose amitriptyline prevents PHN.

## Introduction

Herpes zoster, or ‘shingles’, is characterized by a painful, blistering, unilateral dermatomal rash caused by reactivation of the varicella zoster virus within a dorsal root or cranial sensory ganglion. The estimated lifetime risk of shingles is approximately 30% and the risk and severity of shingles and its complications increases steeply with age, and is higher in individuals who are immunocompromised.^1^

The most common complication of shingles is post herpetic neuralgia (PHN). This chronic neuropathic pain can last for decades and has a major detrimental impact on the quality of life of affected patients.^2,3^ Patients with chronic pain are also at higher risk of other complications, including depression and cardiovascular disease.^4^

PHN is thought to be a direct consequence of the peripheral nerve damage caused by virus reactivation in the ganglion.^5^ The early administration of antivirals reduces the acute pain of shingles but has never been shown to reduce the incidence of PHN.^6^ Other attempts to prevent PHN using either corticosteroids, gabapentinoids or duloxetine have proven ineffective.^7–9^

Amitriptyline may prevent nerve damage by binding to nerve growth factor receptors,^10^ a mechanism distinct from its noradrenaline reuptake inhibition used in the treatment of chronic pain. In 1997, a single-centre study of 80 patients reported that low-dose amitriptyline halved the incidence of PHN,^11^ but these findings have never been replicated in a large, adequately powered, clinical trial.

### Aim

To determine the clinical and cost-effectiveness of prophylactic low-dose amitriptyline for the prevention of PHN in patients diagnosed with shingles.

### Objectives

The primary objective of the trial is to assess the clinical effectiveness of low-dose amitriptyline compared with placebo for the prevention of PHN at 90 days (primary outcome).

Secondary trial objectives are to:

- assess the safety, tolerability and acceptability of amitriptyline used for the prevention of PHN
- compare the impact on shorter (<90 days) and longer-term (up to 12 months) outcomes of pain, quality of life, mental wellbeing and frailty

The nested health economic study will:

- evaluate the cost-effectiveness of low-dose amitriptyline compared with placebo in terms of cost per case of PHN prevented and cost per quality-adjusted life year (QALY) gained, at 90 days and 12 months.

The nested qualitative study will:

- support and optimize delivery of the trial
- explore acceptability and perceived effectiveness of the intervention during the trial, to aid interpretation and implementation of the trial findings

### Trial design

This is a multicentre, individually randomized, parallel-group, pragmatic, placebo-controlled superiority trial [type A Clinical Trial of an Investigational Medicinal Product (CTIMP)] with health economic analysis and nested qualitative study. The internal pilot ran from 1 April 2022 to 31 December 2022, and participant recruitment is expected to finish by April 2025.

## Patients and methods

### Study setting

Primary care [general practitioner (GP) surgeries] in 10 Regional Research Delivery Networks in England.

### Eligibility criteria

Table 1 gives the detailed inclusion/exclusion list. The key inclusion criteria are patients with shingles aged ≥50 years, presenting with their first or second episode of shingles, 6 days or less since rash onset and not already taking (and with no contraindication to taking) amitriptyline 10–30 mg.

**Table 1:** Participant inclusion and exclusion criteria

Inclusion criteria	Exclusion criteria
Adults aged ≥50 years	Third or more episode of herpes zoster
Clinical diagnosis of herpes zoster	Known adverse reaction to amitriptyline or contraindications (monoamine oxidase inhibitors)
Rash onset <144 h	Current/recent (within previous 2 weeks) use of a tricyclic antidepressant
	Known prolonged QT interval or concomitant drugs that prolong the QT interval
	Suicidal ideation
	Known heart block
	Recent (within 4 weeks) myocardial infarction
	Immunosuppression^a^
	Known significant bradycardia
	Uncompensated heart failure
	Hyperthyroidism
	Severe liver disease
	Phaeochromocytoma
	Urinary retention
	Current or planned (in the next 3 months) pregnancy or breastfeeding
	Currently (or recently, within the previous 4 months) enrolled in another CTIMP
	Inability to provide informed consent and complete study assessments/questionnaires

^a^Immunosuppression is defined as: disease or treatment, including patients undergoing chemotherapy leading to immunosuppression; patients undergoing radical radiotherapy; recipient of solid organ, bone marrow or stem cell transplants; HIV infection; haematological malignancy, including leukaemia, lymphoma and myeloma; genetic disorders affecting the immune system (e.g. IRAK-4 deficiency, NEMO deficiency syndrome, complement disorder, severe combined immunodeficiency); immunosuppressive or immunomodulating biological therapy, including anti-tumour necrosis factor, alemtuzumab, ofatumumab, rituximab; protein kinase inhibitors or PARP inhibitors; sparing agents such as cyclophosphamide and mycophenolate mofetil; systemic steroids for > 1 month at a dose equivalent to prednisolone at 20 mg or more daily for adults.

### Participant recruitment

Identification of potentially eligible patients by participating GP surgeries is supported in two ways:

- computer ‘pop-ups’ that appear when a relevant diagnostic code is entered
- regular (2–3 times weekly) electronic medical record (EMR) searches for potentially eligible patients

Clinicians who are able to screen and refer patients into the study are ‘first-contact’ healthcare professionals who diagnose shingles and prescribe amitriptyline to patients as part of their normal practice, have watched an ‘AmiTriptyline for the prevention of post-HErpetic NeuralgiA’ (ATHENA) training video and have been added to their local delegation log.

We have adopted a ‘deferred recruitment’ approach,^12^ where the clinician’s role is to only introduce the study and confirm interest and eligibility. The research team contact interested and eligible patients to receive consent as soon as possible.

Referring clinicians submit the name and contact details of eligible and interested patients via an online form that collects age, sex assigned birth and days since rash onset from all patients. For eligible and interested patients, the form also asks for patient-reported average shingles-related pain in the last 24 h (0=‘no pain’; 10=‘pain as bad as you can imagine’), dermatome affected, shingles vaccination history (yes or no/don’t know as binary) and, if an antiviral has been prescribed, which one (including dose and duration). If the patient is not eligible and/or not interested, the clinician is asked to give a reason.

The study medication is prescribed electronically by a clinician on the research team. However, if they believe the eligibility criteria have not been met, the patient and GP surgery are notified and randomization does not take place.

### Intervention

Participants are randomized 1:1 to receive amitriptyline 10 mg or placebo tablets. The placebo is formulated and manufactured to match the active tablet. Study medication is dispensed and dispatched by a central dispensing pharmacy to participants’ home addresses or GP surgeries for collection.

**Figure 1:**
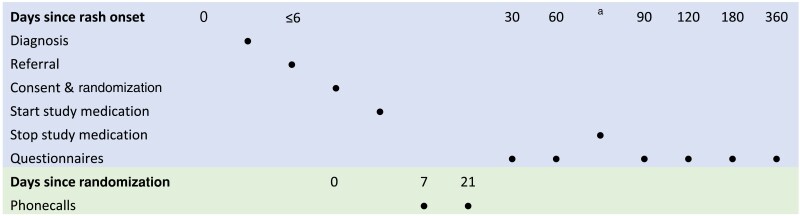
Schema of participant timeline. ^a^Seventy days from starting medication or 80 days from randomization, whichever comes first. All days are counted from date of rash onset, except for research team contact at approximately days 7 and 21 postrandomization.

A medication information leaflet explains how to self-­titrate over the first 2 weeks, a reminder of potential side-­effects, what to do about other medication and how to seek help. Participants are told to start taking one tablet daily, increasing by one tablet every 5–7 days up to a maximum dose of three tablets daily, as tolerated. No additional measures are undertaken to enhance adherence. Participants or their clinicians can choose to discontinue the study medication at any time. Participants are asked to stop their medication 80 days after rash onset or 70 days from starting the study medication, whichever comes sooner, to ensure a ‘­washout’ period of 10 days (the half-life of amitriptyline after a single dose is approximately 24 h)^13^ before the collection of primary outcome information at 90 days from rash onset (Figure 1).

Initially, 1 bottle (70 tablets per bottle) is sent, which covers approximately 28 days. Around 3 weeks postrandomization, one or two further bottles (as required) are sent for the remaining treatment period. Participants are also sent a card to carry, which states that they may be taking active medication during the intervention period and how to unmask in an emergency or report a serious adverse event.

Treating clinicians are not permitted to prescribe amitriptyline to participants during the primary outcome period but can start/stop other medications as per usual care, including treatments for shingles-related pain, as long as they are compatible with amitriptyline.

### Outcomes

The primary outcome is the presence/absence of PHN at 90 days after rash onset, using a cutoff of ≥3/10 on the worst pain in last 24 h (on an 11-point numerical rating scale; where 0=‘no pain’ to 10=‘pain as bad as you can imagine’) item of the Zoster Brief Pain Inventory (ZBPI).^14^ The secondary outcomes are listed in Table 2.

**Table 2:** Schedule of enrolment, interventions and assessments

	Enrolment		Days after randomization (R)				Days after rash onset						
	0–6 days after rash onset	Baseline appointment	R + 1 to R + 6	R + 7	R + 21	R + 28	30	60	80	90	120	180	360^a^
Enrolment													
Eligibility screening and referral^b^	●												
Demographics		●											
Interventions													
Antiviral medication^c^	○												
Study medication^c^				[––––––––––––––––––––––––––––––––]									
Assessments													
ZBPI		●					●	●		●	●	●	●
EQ-5D-5L		●								●		●	●
PHQ-9		●								●		●	●
GAD-7		●								●		●	●
TFI		●								●		●	●
Medication use				●	●		●	●		●			
Adverse events^d^				●	●		●	●		●	●	●	●
Masking^e^							●	●		●			
Participant-reported resource use^f^										●		●	●
GP review						○							

Filled circles indicate all participants; empty circles denote ‘optional’, according to clinical need. EQ-5D-5L, 5-level EQ-5D (generic quality of life);^15^ GAD-7, General Anxiety Disorder 7-item scale;^16^ GP, general practitioner; PHQ-9, Patient Health Questionnaire-9;^17^ TFI, Tilburg Frailty Indicator;^18^ ZBPI, Zoster Brief Pain Inventory (shingles-related pain and interference with life items).^14^  ^a^Participants randomized by 31 March 2024 take part in the trial for 12 months; any patients randomized between 1 April 2024 and 31 January 2025 are followed-up for 180 days, and from 1 February 2025 for 90 days. ^b^Eligibility screening and referral via an online form by a trained clinician at GP surgery, which collects age, sex and days since rash onset. The screening form additionally asks reason for nonreferral. The referral form collects data on: shingles vaccination status; whether an antiviral was prescribed and it so what type, dose and duration; and average shingles related pain (from 0=’no pain’ to 10=‘pain as bad as you can imagine’) in the past 24 h. ^c^Duration/how many days after rash onset varies: antiviral medication is typically prescribed for 1 week; baseline appointments are conducted and medication is started as soon as possible after referral; participants are supplied a with a maximum of 70 days of study medication, with instructions to stop by day 80 after rash onset. ^d^Adverse events were reported by patients and clinicians. ^e^Masking was done using the Bang Blinding Index. ^f^Participant-reported National Health Service resource use, using ModRUM,^20^ and from the electronic medical records of patients who gave consent.

### Participant timeline, data collection methods and retention

Participants take part in the trial for 12 months (randomized by 31 March 2024), 6 months (randomized between 1 April 2024 and 31 January 2025) or 3 months (randomized on 1 February 2025 or later).

Most outcome data are collected by participant-­completed online or paper questionnaires, according to patient preference, with text, email or telephone reminders. All participants are asked to complete questionnaires at baseline and at 30, 60 and 90 days, and for participants with longer follow-up, at 120, 180 and 360 days. All questionnaires are dated from shingles rash onset (Figure 1).

In addition to questionnaire reminders, to encourage engagement participants are sent regular participant newsletters. In recognition of the time participants dedicate to the study, participants are sent £10 vouchers at baseline and on completion of the 90-, 180- and 360-day surveys.

Participants are contacted by a member of the research team around 7 days and 21 days after randomization. These contact points are also to support and monitor self-titration, and collect data on medication use and adverse events. If the participant or research nurse have any queries or concerns about safety, participants are offered a review with their GP surgery.

With permission, after follow-up is complete, we will extract data from the patient’s EMR for data on use of healthcare resources.

### Sample size

Our sample size of 846 is based on a clinically important proportionate reduction of at least 45% in the incidence of PHN (based on the study by Bowsher^11^) at 90 days with 90% power, assuming 20% incidence of PHN in the placebo control group and a 20% loss to follow-up.

### Allocation

Participants are randomized in a 1:1 ratio to receive amitriptyline or placebo, stratified by recruiting centre and minimized on age deciles (50–59 years, 60–69 years, etc.), sex (male or female), average shingles pain at entry to study (cutoff ≥3 on a 0–10 numerical rating scale for average pain in the last 24 h) and shingles vaccination history (yes or no/don’t know as binary).

The randomization sequence is generated by the company Sealed Envelope™ using their secure online randomization system.^21^

The pharmacy uses the randomization number to determine the relevant allocation from the randomization list, then select the next bottle of investigational medicinal product (either amitriptyline or placebo) accordingly and record the kit code.

The participant’s GP surgery is sent a letter informing them that their patient is taking part in the trial, along with a copy of the consent form. The practice is asked to record in the patient’s EMR their participation and that they may be taking amitriptyline 10–30 mg.

### Masking

Participants, treating clinicians and the research team are masked to treatment allocation. If there is a clinical need for emergency unmasking, the participant’s treating clinician can telephone the pharmacy’s 24-h service.

The junior trial statistician is unmasked (for the purpose of preparing the closed reports for the Data Monitoring Committee), as are the study dispensing pharmacists (to be able to issue the allocated study medication). The unmasked randomization codes are held by the junior statistician and study pharmacy only. Treatment codes will only be released to the research team once the trial database has been locked. After this, participants and their GPs will be informed of their allocation.

### Data management

Self-completed online questionnaires are entered directly into a REDCap database. Questionnaires completed on paper will be manually entered into the REDCap database by the research team. The quality of the trial data will be monitored throughout the trial.

### Statistical methods

Analysis and presentation of the trial data will be in accordance with the CONSORT^22^ and CONSORT PRO^23^ guidelines. A full analysis plan will be completed and made publicly accessible prior to analysis.

Baseline patient characteristics will be compared using descriptive statistics. Meaningful differences between the groups at baseline will inform subsequent sensitivity analyses, adjusting for imbalances.

The primary statistical analyses will be conducted on an intention-to-treat principle. The primary analysis will use logistic regression to estimate the odds ratio of PHN at 90 days after rash onset comparing the intervention and control group, adjusting for variables used in the randomization.

Repeated measures analyses will be conducted on the secondary outcomes measured at multiple follow-up ­timepoints to examine the effect of the intervention over time. These will involve logistic regression models for binary outcomes and linear regression models for continuous outcomes. Descriptive analysis of safety endpoints will be presented according to randomized group.

Because this is a pragmatic study, where participants can self-titrate the study medication as tolerated, there are no strict adherence criteria. However, participants will be included in ‘per-protocol’ analyses if they start medication within 12 days of rash onset, and report taking it every day until their scheduled stop date. Complier average causal effect analysis will investigate the efficacy of the intervention for comparison with the primary intention-to-treat effect estimate, as well as explore whether there are patient and illness characteristics associated with adherence. Sensitivity analyses will assess the robustness of the primary analysis to the impact of missing data on the primary analysis; adjustment for variables with marked imbalance at baseline; and antiviral treatment.

Subgroup analyses will examine whether the effect of the intervention on pain outcomes differs according to time from rash onset to starting treatment; daily use and total dose; and whether it is the first or second episode of shingles. Subgroup analyses will be performed by incorporating a treatment group–subgroup interaction term in the appropriate regression model. Testing will be done using the likelihood ratio test.

### Monitoring

For the duration of the study, the independent Data Monitoring Committee (DMC) and Trial Steering Committee will meet regularly and monitor the study, to ensure it is conducted according to good research practice.

We will regularly monitor the characteristics of screened, referred and randomized patients; and data completeness, participant retention and the proportion of participants reporting PHN and adverse events. In closed meetings with the DMC, the unmasked junior statistician will provide these data by allocation.

An annual Development Safety Update Report is submitted to the Medicines and Healthcare products Regulatory Authority (MHRA) until the end of the trial is declared.

### Harms

It is anticipated that most adverse events will be detected via the questionnaires administered at the follow-up timepoints.

Pharmacovigilance will be carried out in accordance with the guidance set out by the European Commission Detailed Guidance CT-3 2011, and the requirements of the Medicines for Human Use (Clinical Trials) Regulations.

### Auditing

Auditing will be independently undertaken by University Hospitals Bristol and Weston (UHBW) National Health Service (NHS) Foundation Trust on behalf of the sponsor. A Trial Monitoring Plan has been developed by UBHW and agreed by the sponsor, Trial Management Group and chief investigator based on the trial risk assessment.

### Trial registration

The trial registration numbers are EudraCT 2021-001101-78 and ISRCTN14490832.

## Health economics

The primary analyses will present cost-effectiveness (in terms of cost per case of PHN prevented) and cost–­utility analysis from an NHS and personal social services perspective at 90 days. For the cost–utility analysis, QALYs will be estimated using utility scores derived from the 5-level EQ-5D (EQ-5D-5L) and valued by application of the National Institute for Health and Care Excellence (NICE)-recommended value set approach at the time of analysis.^24^ Secondary analysis will consider if the effect is sustained over 12 months.

Resource-use data will be collected from EMR and participant questionnaires, using a ModRUM core healthcare module and bespoke questions covering social care, informal care, personal expenses and usual activities.^20^ The questionnaire and EMR (where feasible to categorize) data will focus on resource use relevant to shingles pain. Further detail will be prespecified in the Health Economics Analysis Plan. Resources will be valued using nationally available sources of unit costs.

Where relevant, incremental cost-effectiveness ratios will be estimated for cases of PHN prevented and QALYs gained. The incremental net monetary benefit will be estimated at willingness-to-pay thresholds of £20 000 and £30 000 per QALY. Statistical methods will deal with skew, baseline imbalance, missingness and sampling uncertainty as appropriate. Cost-effectiveness acceptability curves will show the probability that amitriptyline is the optimal choice over a range of possible values of the ceiling ratio.

## Qualitative study

### Phase 1: support and optimize delivery of the trial

Initially, qualitative work will focus on identifying potentially modifiable barriers to recruitment, along with exploring initial acceptability of the intervention.

The first is done by reviewing a sample of audio-recordings of recruitment conversations undertaken by researchers and other staff as part of the two-step deferred consent process. Summaries of factors identified that may help or hinder recruitment, and how the latter could be modified, are fed back to recruiting staff.

Next, brief (5–20 min) telephone interviews are conducted with up to 20 staff who have been involved in identifying and recruiting patients, and up to 20 patients (within 1 month of them presenting to their GP) who have consented to participate, declined or withdrawn from the trial during the internal pilot phase. These interviews are exploring understanding of the trial; factors that influence recruitment; and views of intervention acceptability.

### Phase 2: acceptability and perceived effectiveness of the intervention

In the main trial phase, qualitative work focuses on understanding acceptability and perceived effectiveness of the intervention, to aid interpretation and implementation of the trial findings.

We are conducting semi-structured telephone interviews with up to 30 patients (to achieve sufficient information power)^25^ at around 2 months postrandomization. Patients are purposefully sampled across intervention and control groups to ensure variation in age, sex, pain severity and adherence. We aim to include patients who stop treatment or drop out of the trial with an exploration of reasons why they drop out.

In interviews, we are exploring patients’ understanding and experiences of the intervention, focusing on perceptions of amitriptyline, experiences of self-titration and alterations in dosage, experiences of side-effects, and impact on willingness to continue treatment and stay in the trial.

Interviews in both phases are audio-recorded and transcribed verbatim. Analysis will be thematic, using a combination of inductive and deductive coding.

## Ethics and dissemination

### Protocol amendments

The sponsor can make a nonsubstantial amendment at any time during a trial. For substantial amendments to the Clinical Trial Authorisation (or the documents that supported the original application) or to the research ethics committee (REC) application (or supporting documents), the sponsor will submit a valid notice of amendment to the MHRA or REC respectively.

### Consent

On receipt of a referral, the potential participant is sent the full Participant Information Leaflet. A researcher arranges a time to speak with them (by telephone, video call or, where necessary, in person), to ensure full understanding of the study and any questions will be answered. Consent will mostly be online,^26^ but for people unable to do this, written consent will be received in person or by post.

### Confidentiality

Information from participants will only be identifiable via a unique study identification number. Participant data will be securely stored in locked cabinets and on University of Bristol servers.

Personal data will not be kept for longer than is required for the purpose for which it has been acquired. Research data will be kept for at least 15 years, when all paper records will be destroyed by confidential means.

Interview data captured on the encrypted audio-recorder will be transferred to a University of Bristol computer as soon as possible after each interview. Audio recordings will be transcribed by university-approved transcription services. Transcripts will be labelled with a study-assigned participant number, edited to ensure the anonymity of respondents and stored securely.

### Ancillary and post-trial care

Prescriptions for amitriptyline after the intervention phase are the responsibility of the participant’s normal clinician, but can only occur after collection of the primary outcome at 90 days after shingles rash onset.

### Dissemination policy

A plan for disseminating the trial results will be developed by the Trial Management Group. Initial findings will be submitted to relevant national and international meetings. Participants will be sent a lay summary and the results of the study will be published in peer-reviewed journals.

## Discussion

While vaccination programmes have implications for the number of potentially eligible patients, data also emphasize the importance of trying to find options to prevent PHN in those individuals who still develop shingles. The shingles vaccine Zostavax^®^ was introduced in the UK in September 2013 with the aim of boosting immunocompetent 70- to 79-year-olds’ pre-existing immunity to varicella zoster virus. A single dose in adults aged 60 years or older has been shown to reduce the incidence of shingles and PHN by 51.3% and 66.5%, respectively,^27^ with evidence of sustained effectiveness up to 8 years after vaccination.^28^ During its first 5 years, it is estimated to have saved 40 500 GP consultations for shingles, including 8700 fewer for PHN.^29^ Since September 2023, eligibility criteria have been broadened (to include immunocompromised patients aged 50 years and older, with the lower age limit for immunocompetent patients going down to 60 years in September 2028) and individuals are now offered two doses of Shingrix^®^. The effectiveness of one and two doses of Shingrix^®^ in people aged >65 years is estimated to be 56.9% and 70.1%, respectively. Two-dose vaccine effectiveness against postherpetic neuralgia was 76.0%.^30^

This study was commissioned to test the findings from the trial by Bowsher.^11^ While Bowsher’s study suggested that early, low-dose (25 mg) amitriptyline in patients with shingles reduced the prevalence of PHN by 45%, the study was small (80 patients), methodologically limited and poorly reported. Consequently, prophylactic prescribing of amitriptyline to prevent PHN is neither recommended in guidelines nor widely used. Amitriptyline is a cheap, readily available medicine that is already used in the treatment of established PHN, but its potential associated harms mean this definitive trial is needed before the routine prophylactic use of amitriptyline can be recommended.

Recruiting patients with incident conditions into a CTIMP in primary care is challenging. To facilitate the process, we initially relied just on EMR ‘pop-ups’ but some sites favour regular searches instead of, or alongside, this. Initially, only GPs were able to screen and refer patients but, reflecting clinical practice, this was broadened to all ‘first-contact’ healthcare professionals, including, but not limited to, advanced nurse practitioners and clinical pharmacists. We originally sought to complete participant recruitment over 18 months from 120 GP sites. However, because the number of eligible patients has been lower than expected, we extended recruitment to 37 months from over 120 group practices (>300 GP surgeries/sites). In order to complete the trial in a timely fashion, a consequence of this extension to recruitment was a reduction in the follow-up period from 360 to 180 days for patients randomized between 1 April 2024 and 31 January 2025, and 90 days for patients randomized on 1 February 2025 or later.

We aim for patients to start the study medication as soon as possible and for participants in the intervention group to take amitriptyline 30 mg daily for up to 70 days. However, because of clinical service constraints it is not always possible for participants to start taking tablets within 72 h of rash onset (as per Bowsher’s trial)^11^ and because of the uncertainty as to the minimum amitriptyline dose required to be effective, and to reduce the risk of stopping due to side-effects, participants are told to titrate their dose up to three tablets daily over the first 2 weeks as tolerated. Both of these aspects reflect current clinical care in the NHS, helping to make the findings generalizable to this setting.

We capture shingles-related pain using the most widely used measure, the ZBPI. While there is no universally agreed definition of PHN, we adopted the most commonly used definition of ≥3/10 in the affected dermatome, 90 days following rash onset.^31^ We originally specified that the primary outcome would be based on average pain. However, most studies of shingles/PHN have defined PHN on basis of ‘worst pain ≥3/10’. ‘Worst pain’ has also been shown to be the most reliable of the parent BPI scores,^32,33^ and to correlate mostly strongly with interference with mood, sleep and everyday activities.^32^ In addition, feedback from participants in the nested qualitative study and members of our patient advisory group was that worst pain is an easier concept for patients than average pain, especially for a pain that is commonly paroxysmal. For these reasons, we changed the definition of PHN from using a cutoff of ≥3/10 on numerical rating scale average pain in last 24 h to worst pain in the last 24 h. Planned secondary analyses will capture any effects on the other ZBPI measures, as well as impact on mood, quality of life and frailty. We may also be able to assess whether we see any sign of a benefit of amitriptyline in ameliorating the acute pain of shingles at the 30- and 60-day timepoints.

## Data Availability

Anonymized research data will be stored securely for future analysis on a research data facility. Requests for access to data must be via a written confidentiality and data-sharing agreement, which will cover limitations of use, transfer to third parties and storage. Anyone applying for use of the data will be scrutinized for appropriate eligibility.
